# Synergistic Antibacterial Effects of Nanoparticles Encapsulated with *Scutellaria baicalensis* and Pure Chlorhexidine on Oral Bacterial Biofilms

**DOI:** 10.3390/nano6040061

**Published:** 2016-04-07

**Authors:** Ken Cham-Fai Leung, Chaminda Jayampath Seneviratne, Xuan Li, Ping Chung Leung, Clara Bik San Lau, Chi-Hin Wong, Ka Yan Pang, Chun Wai Wong, Elaine Wat, Lijian Jin

**Affiliations:** 1 Department of Chemistry, Institute of Creativity, and Partner State Key Laboratory of Environmental and Biological Analysis, The Hong Kong Baptist University, Kowloon, Hong Kong, China; cfleung@hkbu.edu.hk (K.C.-F.L.); hinciwong@gmail.com (C.-H.W.); 2 Faculty of Dentistry, National University of Singapore, 11 Lower Kent Ridge Road, Singapore City 119083, Singapore; chaminda_jayampath@nuhs.edu.sg; 3 Faculty of Dentistry, The University of Hong Kong, 34 Hospital Road, Hong Kong, China; lixuanlwj@hotmail.com (X.L.); nisdabendan@gmail.com (K.Y.P.); 4 Institute of Chinese Medicine and Partner State Key Laboratory of Phytochemistry and Plant Resources in West China, The Chinese University of Hong Kong, New Territories, Hong Kong, China; pingcleung@cuhk.edu.hk (P.C.L.); claralau@cuhk.edu.hk (C.B.S.L.); cwwong_eric@cuhk.edu.hk (C.W.W.); elaine.wat@cuhk.edu.hk (E.W.)

**Keywords:** nanotechnology, *Scutellaria baicalensis*, traditional Chinese medicine, chlorhexidine, oral biofilms, antimicrobials

## Abstract

*Scutellaria* *baicalensis* (SB) is a traditional Chinese medicine for treating infectious and inflammatory diseases. Our recent study shows potent antibacterial effects of nanoparticle-encapsulated chlorhexidine (Nano-CHX). Herein, we explored the synergistic effects of the nanoparticle-encapsulated SB (Nano-SB) and Nano-CHX on oral bacterial biofilms. Loading efficiency of Nano-SB was determined by thermogravimetric analysis, and its releasing profile was assessed by high-performance liquid chromatographyusing baicalin (a flavonoid compound of SB) as the marker. The mucosal diffusion assay on Nano-SB was undertaken in a porcine model. The antibacterial effects of the mixed nanoparticles (Nano-MIX) of Nano-SB and Nano-CHX at 9:1 (*w*/*w*) ratio were analyzed in both planktonic and biofilm modes of representative oral bacteria. The Nano-MIX was effective on the mono-species biofilms of *Streptococcus* (*S.*) *mutans*, *S. sobrinus*, *Fusobacterium* (*F.*) *nucleatum*, and *Aggregatibacter* (*A.*) *actinomycetemcomitans* (MIC 50 μg/mL) at 24 h, and exhibited an enhanced effect against the multi-species biofilms such as *S. mutans*, *F. nucleatum*, *A. actinomycetemcomitans*, and *Porphyromonas* (*P.*) *gingivalis* (MIC 12.5 μg/mL) at 24 h that was supported by the findings of both scanning electron microscopy (SEM) and confocal scanning laser microscopy (CLSM). This study shows enhanced synergistic antibacterial effects of the Nano-MIX on common oral bacterial biofilms, which could be potentially developed as a novel antimicrobial agent for clinical oral/periodontal care.

## 1. Introduction

Periodontal disease is a major global oral health burden and severe periodontitis accounts primarily for multiple tooth loss in the adult population worldwide [1,2]. It badly affects oral health and also significantly links with systemic diseases such as diabetes mellitus and cardiovascular disease [2,3]. Periodontal disease results from aberrant and exaggerated immuno-inflammatory response to pathogenic plaque biofilms [4]. Therefore, various anti-biofilm and host modulatory therapy have been tested for controlling periodontal disease [5]. Chlorhexidine (CHX) has long been recognized as the golden standard of chemical plaque control for oral healthcare products. CHX gluconate-containing mouthwash is a common chemical plaque control measure [6]. However, there are certain side effects associated with CHX containing mouthwash, such as staining on the teeth, unpleasant taste, allergic reactions, and occasionally numbness of the tongue [7,8]. In addition, CHX is a classical disinfectant agent which kills all the bacteria without discriminating harmful pathogens. It might also be toxic to the host cells [9]. This caveat has prompted researchers to look into alternative anti-biofilm and host modulating agents and approaches.

*Scutellaria* *baicalensis* (SB) is a traditional Chinese medicine frequently used for treating infectious and inflammatory diseases for thousands of years [10]. Chinese literature reports that it has been used to manage periodontal disease [11]. Previous studies have confirmed that baicalin, a flavonoid compound isolated from SB, possesses marked anti-inflammatory, antioxidative, and immunomodulating effects [12,13,14,15]. It exhibits protective effect on the development of experimental periodontitis [12] and benefits for controlling periodontal disease [13,16]. We have further shown that baicalin could modulate *P. gingivalis* lipopolysaccharide-induced immuno-inflammatory response in oral epithelia [14]. Interestingly, recent studies indicate that SB has potent antibacterial effects on oral pathogens [17], and it could indeed inhibit bacterial quorum sensing activity [18]. A recent study indicates that the combined use of another compound baicalein present in SB with antibiotics has synergistic effects against oral bacteria [19].

Nano-encapsulation technique has currently become an exciting new approach to the delivery of biologically active compounds and agents more effectively at specific biological niches [20,21]. Hence, nanotechnology has received great attention for developing new healthcare products and drugs [22]. Recently, our team has successfully synthesized a novel mesoporous silica nanoparticle (MSN)-encapsulated chlorhexidine (Nano-CHX), which demonstrates potent antibacterial effects on oral biofilms [23]. It has also been shown that nanoparticle-encapsulated plant extracts enhance the biological effectiveness via bioavailability and fast penetration with less cytotoxicity, reduced dosage of the agents, and low costs [24]. Currently, nanotechnology has been increasingly applied in biomedical research and development of novel drug delivery systems. Therefore, nano-encapsulated Chinese herbal extracts or active compounds could enhance their biological effectiveness.

It has been well documented that oral mucosa serves as an attractive and preferred route for systemic delivery of drugs, due to its featured biological structure, easy accessibility, high acceptance by patients [25]. Compared to the skin, the buccal mucosa is far more permeable [26,27], which makes it an instrumental and feasible approach to screening new drugs, assessing drug delivery efficacy, bioavailability, and underlying mechanisms of drug transport as well as refining transbuccal drug delivery. The Franz-Type Diffusion Cells model using porcine oral mucosa has been employed for assay of drug diffusion and absorption [28]. With the similarity in membrane morphology, biological structure and contents, and permeability barrier properties between human and porcine skins and buccal mucosas, this *ex vivo* model has been well employed on investigation of the feasibility of drug delivery and the underlying drug transport mechanism [29,30].

In the present study, we comprehensively investigated the anti-oral biofilm properties of the Nano-MIX of novel MSN-encapsulated SB (Nano-SB) and Nano-CHX, with reference to our recent findings of Nano-CHX alone against oral biofilms [23]. Meanwhile, the loading and releasing profiles of Nano-SB were characterized, and the capacity of both Nano-SB and SB aqueous extract to penetrate oral mucosa was examined using an *ex vivo* model of porcine buccal mucosa. Herein, we find the significant synergistic antibacterial effects of the combined usage of Nano-SB and Nano-CHX at 9:1 (*w*/*w*) ratio against the mixed oral biofilms, such as *S. mutans*, *F. nucleatum*, *A. actinomycetemcomitans*, and *P. gingivalis*.

## 2. Results

### 2.1. Loading and Releasing Profiles of Nano-SB

**Figure 1:**
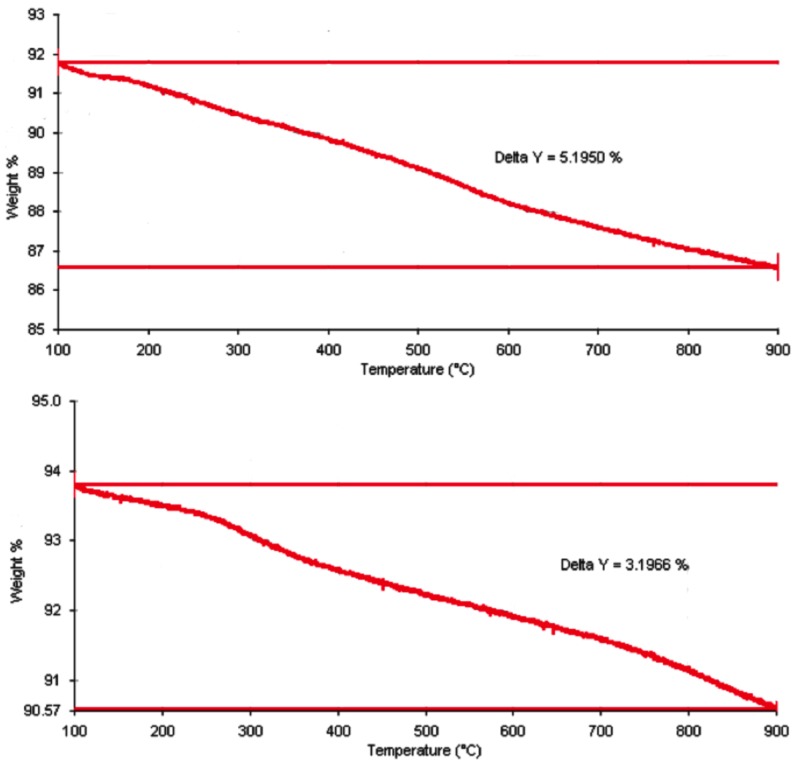
The thermogravimetric analysis on the weight losses of nanoparticle-encapsulated *Scutellaria* *baicalensis* (Nano-SB) (5.2%) (**upper**) and blank nanoparticles (3.2%) (**lower**) between 100 °C and 900 °C.

The aqueous extract of SB was swelled and loaded into MSN. The percentage of SB extract loaded to the nanoparticles was determined by thermogravimetric analyses. As shown in Figure 1, the weight loss of Nano-SB was 5.2% from 100 to 900 °C. As the weight loss of blank nanoparticles was found to be 3.2% during this range of temperature, the SB loading efficiency was calculated to be 2.0% after a background subtraction with the blank nanoparticles.

**Figure 2:**
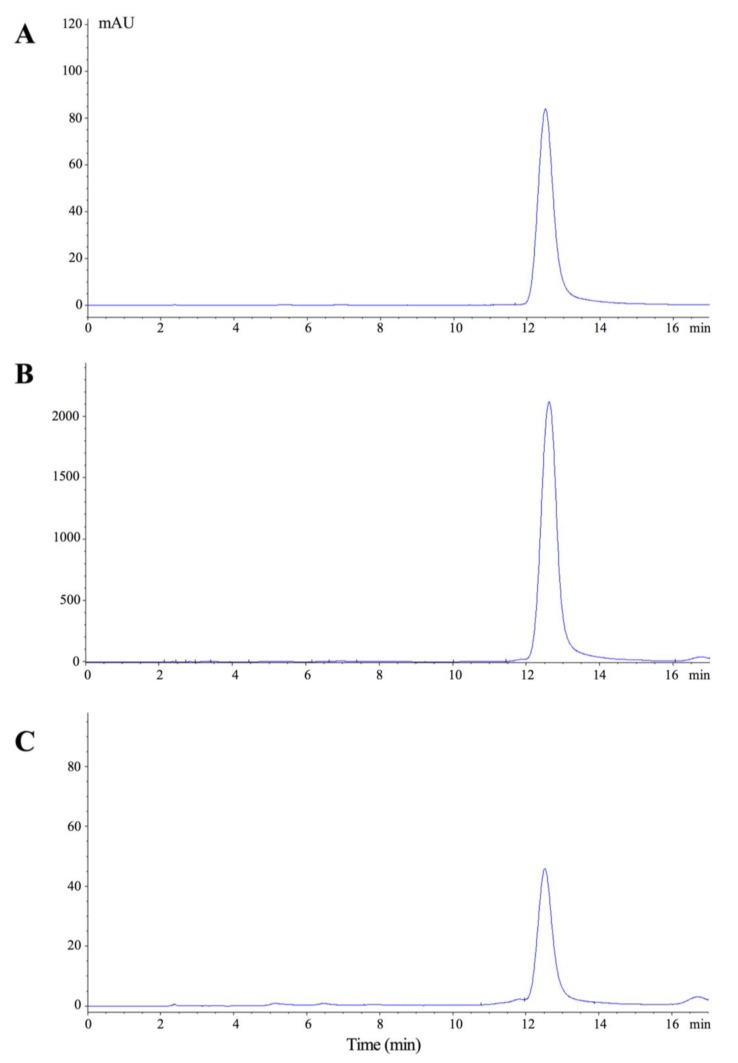
The high-performance liquid chromatography (HPLC) profiles of (**A**) baicalin; (**B**) SB aqueous extract; and (**C**) Nano-SB. Detection was performed at 274 nm, and the retention time of baicalin was assigned at 12.5 min.

The high-performance liquid chromatography (HPLC) analysis of Nano-SB was then performed with reference to baicalin *per se* and SB aqueous extract. The retention time of baicalin, the biomarker of SB, was assigned at 12.5 min. It was noted that the SB content trapped in MSN could be released in water at 24 h, indicating that Nano-SB was able to release baicalin (Figure 2).

### 2.2. The Penetration Capacity of Nano-SB through Porcine Buccal Mucosa

As shown in Table 1, the diffusion experiment found that 14.6% of baicalin, the bioactive component from the Nano-SB aqueous extract, was retained within the mucosal membrane after 2 h of treatment, with reference to 9.8% of SB extract. However, after 6 h of treatment, 46.7% of baicalin penetrated through the oral mucosal membrane, with reference to 72.5% of SB extract. Taken together, the total retention rate (5.8%) and penetration rate (46.7%) of Nano-SB reached 52.5%. Notably, such diffusive and penetrative effects of Nano-SB acted in a relatively slow mode with reference to SB (14.6% *vs.* 48.8% at 2 h; and 52.5% *vs.* 76.0% at 6 h).

### 2.3. Antibacterial Effects of Nano-MIX on Planktonic Bacteria and Mono- and Multi-Species Oral Biofilms

To investigate the synergistic effects of the mixed nanoparticles (Nano-MIX), Nano-SB and Nano-CHX at various ratios were tested in pilot experiments. The 9:1 (*w*/*w*) ratio of Nano-SB and Nano-CHX was determined appropriately, and the Nano-MIX was then prepared for the subsequent experiments. The weight percentages of SB and CHX in the Nano-MIX were 1.80% and 2.02%, respectively. The minimum inhibitory concentration (MIC, µg/mL) values of Nano-MIX on the planktonic mode and mono-species biofilms of selected oral pathogens with reference to the blank nanoparticles are presented in Table 2. Notably, the Nano-MIX treatment for 24 h effectively inhibited the mono-species biofilms of *S. mutans*, *S. sobrinus*, *F. nucleatum*, and *A. actinomycetemcomitans* at a low MIC of 50 µg/mL. The Nano-MIX also inhibited the mono-species biofilms of *Enterococccus* (*E.*) *faecalis* with a relatively high MIC of 200 µg/mL.

**Figure 3:**
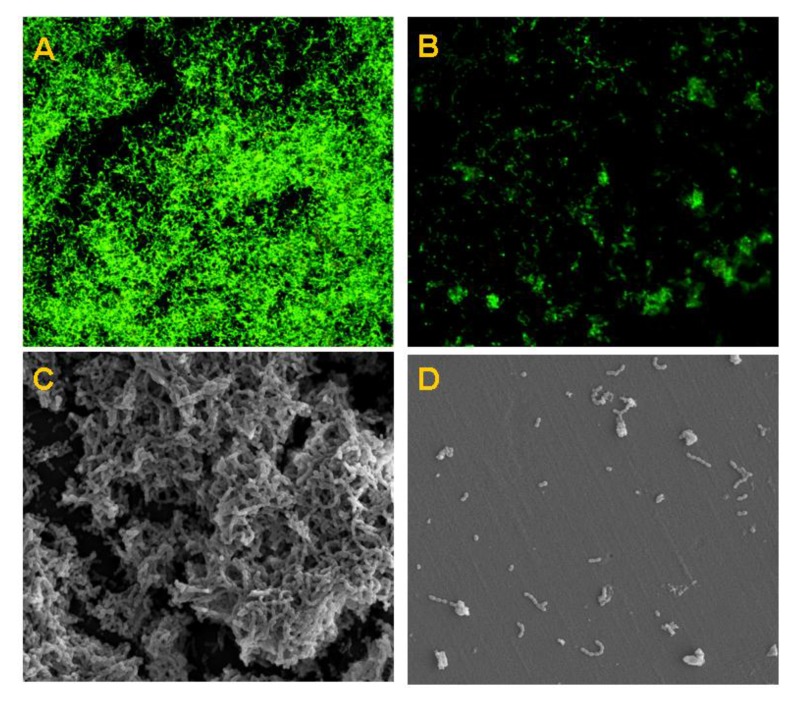
Effect of the Nano-MIX on the multi-species biofilms of *S. mutans*, *F. nucleatum*, *A. actinomycetemcomitans*, and *P. gingivalis* at 24 h. The confocal scanning laser microscopy (CLSM) (**A**,**B**) and scanning electron microscopy (SEM) images (**C**,**D**) showing comparative antibacterial effects of the Nano-MIX treatment (**B**,**D**) on the mixed-species oral biofilms with reference to the blank nanoparticles (**A**,**C**), respectively.

Moreover, we further examined the preventive action of Nano-MIX on the formation of three selected multi-species biofilms at 24 h and 48 h. Interestingly, the Nano-MIX had potent antibacterial effects on the mixed-species biofilms of *S. mutans*, *F. nucleatum*, and *P. gingivalis*; *S. sobrinus*, *F. nucleatum*, and *P. gingivalis*; and *S. mutans*, *F. nucleatum*, *A. actinomycetemcomitans*, and *P. gingivalis*, at low MIC levels of 12.5 μg/mL (24 h) and 50 μg/mL (48 h), respectively (Table 3). The anti-biofilm effect on multi-species biofilms of *S. mutans*, *F. nucleatum*, *A. actinomycetemcomitans*, and *P. gingivalis* at 24 h was further confirmed by both confocal scanning laser microscopy (CLSM) and scanning electron microscopy (SEM). The findings of CLSM (Figure 3A,B) and SEM images (Figure 3C,D) showed that the Nano-MIX treatment markedly affected the multi-species biofilms with few remaining isolated bacterial cells, with reference to the controls treated with blank nanoparticles.

## 3. Discussion

The characters of the MSN used in the present study have been previously described, presenting an average diameter of 140 nm with both pore size and volume of about 2.5 nm and 1.0 cm^3^/g, respectively; the CHX loading efficiency of Nano-CHX is 20.2% [23]. In the present study, the SB loading efficiency of Nano-SB is 2.0%. The loading efficiency of a substrate towards the mesopores with a fixed diameter ~2 nm of the silica nanoparticle is governed by the molecular size and shape, polarity of the substrate, and the solvent and method used in the loading process. Moreover, the HPLC analysis reveals that the Nano-SB enables the containment and release of baicalin, the marker of SB. Hence, the nano-encapsulation and releasing process of SB have been effectively undertaken.

CHX as an essential ingredient in commonly used mouthwash is highly effective for controlling plaque biofilms [6]. However, it has been reported that CHX has some adverse effects like tooth staining and hypersensitive response [7,8]. SB or its active species have been loaded to the magnetic nanoparticles [31,32,33] and chitosan nanoparticles [34], and these studies show that the nanoparticles loaded with SB derivatives exhibit an enhanced drug delivery percentage, increased apoptosis of cancer cells, and a reversal of multidrug resistance. The present study investigated for the first time the synergistic effects of Nano-SB and Nano-CHX on selected oral biofilms. Furthermore, our initial proof-of-concept experiment on the penetration of Nano-SB through porcine oral mucosa assessed the active interactions of Nano-SB with oral mucosa tissue and its compatibility for the practical oral applications. Furthermore, a significantly reduced amount of Nano-CHX in the Nano-MIX with Nano-SB and Nano-CHX at 9:1 (*w*/*w*) ratio would minimize the unwanted effects of CHX for potential clinical benefits. The findings showed that the Nano-MIX treatment for 24 h was relatively more effective against the planktonic mode of *S. sobrinus* (MIC, μg/mL: 50 *vs.* 100), *F. nucleatum* (25 *vs.* 50), and *E. faecalis* (50 *vs.* 100), with reference to Nano-CHX alone, shown in our recent study [23]. Notably, this trend was also observed against mono-species biofilms, as Nano-MIX had a comparatively lower MIC than the Nano-CHX for *S. mutans* (50 *vs.* 100), *F. nucleatum* (50 *vs.* 100), *A. actinomycetemcomitans* (50 *vs.* 100), and especially *S. sobrinus* (50 *vs.* 200). As such, the Nano-MIX exhibited one-fold lower MIC against *S. mutans*, *F. nucleatum*, and *A. actinomycetemcomitans*, and 4-fold lower MIC against *S. sobrinus*.

Furthermore, Nano-MIX also demonstrated more effective preventive actions against the formation of multi-species biofilms of *S. sobrinus*, *F. nucleatum* and *P. gingivalis*, and *S. mutans*, *F. nucleatum*, *A. actinomycetemcomitans* and *P. gingivalis* after 24 h treatment, showing one-fold lower MIC with reference to the Nano-CHX alone (12.5 *vs.* 25 μg/mL), respectively [23]. The anti-biofilm effect on *S. mutans*, *F. nucleatum*, *A. actinomycetemcomitans* and *P. gingivalis* was well supported by the findings of SEM and CLSM images. Similarly, some recent studies have indicated the enhanced antimicrobial activity when pure drugs are incorporated into nanoparticle carriers [35,36]. Hence, nanotherapy may bring clinical benefits to the patients suffering from oral biofilm-induced infections and inflammation (e.g. periodontal disease) in the future, although more studies are warranted to translate the current findings into clinical usage.

The present study indicates the enhanced synergistic antimicrobial effects of the Nano-MIX on common oral bacterial biofilms. Further investigation is highly warranted to refine the protocol for optimal antimicrobial effectiveness. It is potentially possible to develop novel nanoparticle-encapsulated multiple agents such as herbal medicine and pure CHX for better oral and periodontal care.

## 4. Materials and Methods

### 4.1. SB Extract and Nano-CHX

SB aqueous extract was kindly provided by the Hong Kong Premier Concentrated Chinese Herbs Ltd., Kowloon, China, with an extraction yield of 33.3%. Pure chlorhexidine (base form) and other chemicals were purchased from Sigma-Aldrich (St. Louis, MO, USA). MSN and Nano-CHX were prepared as described in our recent study [23].

### 4.2. Thermogravimetric Analysis of SB Loading in MSN

SB extract (50 mg) was dissolved in ethanol (5 mL). The undissolved extracts or compounds were filtered off using a membrane with a pore size of 0.2 micron. The concentration of filtrate was fixed at 7 mg/mL. 50 mg of MSN were then added into 4 mL of the filtrate. After incubation at room temperature for 24 h, the mixture was centrifuged. The SB aqueous extract-loaded MSNs (Nano-SB) were collected by membrane filtration. Its loading effeminacy was determined with reference to blank MSNs through thermogravimetric analyses by the Perkin Elmer TGA-6 (Waltham, MA, USA), according to an established protocol [20].

### 4.3. High-Performance Liquid Chromatography (HPLC) Analyses

The chromatographic profiles of baicalin (a biomark of SB), the SB aqueous extract, and the released Nano-SB were analyzed by a HPLC System, Hewlett Packard Agilent 1100 series equipped with G1329A ALS Autosampler and G1315A Diode Array Detector (Agilent Technologies, Waldbronn, Germany). The solvents were then pre-filtered with the Millipore filter disk (0.45 μm) (Millipore, Darmstadt, Germany) and de-gassed. A gradient elution was undertaken using the mobile phases A (methanol) and B (water/phosphoric acid 99.8/0.2 *v*/*v*). The elution was carried out with a gradient procedure as follows: 0–8 min, 45% B; 8–20 min, from 45% B to 48% B. The flow rate was set at 1.0 mL/min, and the detection was made at 278 nm for baicalin. Ten microliters of each sample were injected into the Ultrasphere ODS C-18 column (Beckman Instrument Inc., Fullerton, CA, USA) after filtration via a filter disk (0.45 μm). The baicalin in the SB aqueous extract and released Nano-SB was then identified by comparing the retention times. The system was monitored by a PC with the 32 Karat Software (Beckman Instrument Inc., Fullerton, CA, USA) for data collection, integration, and analysis.

### 4.4. Preparation of Porcine Buccal Mucosal Membranes and Diffusion Assays

Porcine buccal mucosal membranes were used for the *in vitro* trans-buccal mucosal experiments, to determine the penetration profile of SB and Nano-SB. Fresh porcine heads were obtained from commercial butcher supplier. They were then immediately dissected, and the buccal mucosal membrane was freshly isolated from porcine oral cavity following standard protocol as previously described [25]. In brief, full thickness of the mucosal membrane was cut and liberated from the underlying cartilage using a scalpel. Any adhering subcutaneous fats and tissues were carefully removed. The membrane was then cut into 1.77 cm^2^ sections and stored at −80 °C until further usage.

The diffusion experiments were performed using a diffusion cell system (Taiping Business Mansion, Nanjing, China), consisting of six diffusion cells, diffusion cell drive, and circulating water bath for control of diffusion cells temperature as previously described [37]. Each diffusion cell included a donor and receiving chamber, with a magnetic stirrer at the bottom to ensure thorough mixing of the sample solution at the receiving chamber. The diffusion cell exhibited a diffusion-available surface area of 1.77 cm^2^. Phosphate buffered saline (PBS) (Invitrogen, San Diego, CA, USA) was used as the diffusion medium, with constant stirring at 600 rpm. Temperature was maintained at 37 ± 0.5 °C to ensure all membranes was kept at approximately 32 °C throughout the experiment. Prior to use, all porcine buccal mucosal membranes were soaked in pre-warmed PBS for 5 min to allow hydration of the membranes. All membranes were then carefully placed on top of each of the diffusion cells, avoiding the presence of air bubbles between the membranes and buffer solution. Donor chambers were placed on top of the membranes, and the samples were applied on top of the membranes. All diffusion cells were clipped tightly to avoid evaporation. To determine the amount of baicalin retained in the membrane, it was extracted by soaking into 2 mL of methanol followed by sonication for 1 h; for determining the amount of baicalin diffused through the membrane, the receiver medium was withdrawn at determined intervals. All samples were then analyzed by HPLC.

### 4.5. Anti-Biofilm Properties of the Nano-MIX (Nano-SB and Nano-CHX at 9:1 w/w)

Representative oral bacteria including *S. mutans* (ATCC 35668), *S. sobrinus* (ATCC 33478), *F. nucleatum* (ATCC 25586), *A. actinomycetemcomitans* (ATCC 43718), *E. faecalis* (ATCC 29212), and *P. gingivalis* (ATCC 33277), derived from the archival collection at the Centralized Research Laboratory of the Faculty of Dentistry, The University of Hong Kong, were used in the present study. The MIC of planktonic bacteria was assessed, and mono- and mixed-species biofilms were prepared according to our established procedures [23]. The mixed-species biofilms were made under identical experimental conditions and then tested on the anti-biofilm effects of the nanoparticles at 24 h and 48 h, including (i) *S. mutans*, *F. nucleatum* and *P. gingivalis*; (ii) *S. sobrinus*, *F. nucleatum* and *P. gingivalis*; and (iii) *S. mutans*, *F. nucleatum*, *A. actinomycetemcomitans* and *P. gingivalis*. In separate experiments, biofilms were formed in 1 × 1 cm sterilized polystyrene coupons (IWAKI, Tokyo, Japan). The blank silica nanoparticles were employed as the controls. The multi-species biofilm samples of *S. mutans*, *F. nucleatum*, *A. actinomycetemcomitans* and *P. gingivalis* were subjected to further assessments by both SEM (Hitachi, Tokyo, Japan) and CLSM (Olympus, Tokyo, Japan) at 24 h as previously described [23], in order to confirm the *in vitro* findngs.

### 4.6. Statistical Analysis

Student’s *t*-test or analysis of variance (ANOVA) as appropriate was employed by using the statistical package for the social sciences (SPSS) Statistics (Version 21.0, IBM Corp. Armonk, NY, USA) to determine the statistical significance of MIC between the groups of planktonic and biofilm modes of the bacteria tested, as well as the different time points in the experiments.

## Figures and Tables

**Table 1:** Penetration percentage of SB aqueous extract and Nano-SB during the diffusion cell experiment after treatments of 2 h or 6 h.

Duration of Treatment	Sample	Original Amount of Baicalin (µg)	Baicalin Retained within Mucosa (%)	Baicalin Penetrated to Receiver Chamber (%)
2 h	SB	927	9.8%	39.0%
	Nano-SB	20	14.6%	0.0%
6 h	SB	927	3.5%	72.5%
	Nano-SB	20	5.8%	46.7%

**Table 2:** The minimal inhibitory concentration (MIC, μg/mL) of the mixed nanoparticles (Nano-MIX) of Nano-SB and nanoparticle-encapsulated chlorhexidine (Nano-CHX) at 9:1 (*w*/*w*) ratio against the planktonic mode and mono-species biofilms of selected oral bacteria at 24 h.

Stain	Planktonic Mode	Mono-Species Oral Biofilms
*S. mutans*	50	50
*S. sobrinus*	50	50
*F. nucleatum*	25	50
*A. actinomycetemcomitans*	50	50
*E. faecalis*	50	200 *

* Significant difference from the planktonic mode, *p* < 0.05.

**Table 3:** The minimal inhibitory concentration (MIC, μg/mL) of the mixed nanoparticles (Nano-MIX) of Nano-SB and nanoparticle-encapsulated chlorhexidine (Nano-CHX) at 9:1 (*w*/*w*) ratio against the selected multi-species biofilms of oral bacteria at 24 h and 48 h.

Multi-Species Biofilms	24 h	48 h
*S. mutans*, *F. nucleatum* and *P. gingivalis*	12.5	50 *
*S. sobrinus*, *F. nucleatum* and *P. gingivalis*	12.5	50 *
*S. mutans*, *F. nucleatum*, *A. actinomycetemcomitans* and *P. gingivalis*	12.5	50 *

* Significant difference from 24 h, *p* < 0.05.

## Abbreviations

The following abbreviations are used in this manuscript:

SB	*Scutellaria baicalensis*
Nano-CHX	Nanoparticle-encapsulated chlorhexidine
Nano-SB	Nanoparticle-encapsulated SB
HPLC	High-performance liquid chromatography
Nano-MIX	Nano-SB and Nano-CHX
SEM	Scanning electron microscopy
CSLM	Confocal scanning laser microscopy
MSN	Mesoporous silica nanoparticle
PBS	Phosphate buffered saline
MIC	Minimum inhibitory concentration
